# Deep RNA-Seq to Unlock the Gene Bank of Floral Development in *Sinapis arvensis*

**DOI:** 10.1371/journal.pone.0105775

**Published:** 2014-09-05

**Authors:** Jia Liu, Desheng Mei, Yunchang Li, Shunmou Huang, Qiong Hu

**Affiliations:** Key Laboratory of Biology and Genetic Improvement of Oil Crops, Ministry of Agriculture, Oil Crops Research Institute of the Chinese Academy of Agricultural Sciences, Wuhan, Hubei, People’s Republic of China; Key Laberatory of Horticultural Plant Biology (MOE), China

## Abstract

*Sinapis arvensis* is a weed with strong biological activity. Despite being a problematic annual weed that contaminates agricultural crop yield, it is a valuable alien germplasm resource. It can be utilized for broadening the genetic background of Brassica crops with desirable agricultural traits like resistance to blackleg (*Leptosphaeria maculans*), stem rot (*Sclerotinia sclerotium*) and pod shatter (caused by FRUITFULL gene). However, few genetic studies of *S. arvensis* were reported because of the lack of genomic resources. In the present study, we performed *de novo* transcriptome sequencing to produce a comprehensive dataset for *S. arvensis* for the first time. We used Illumina paired-end sequencing technology to sequence the *S. arvensis* flower transcriptome and generated 40,981,443 reads that were assembled into 131,278 transcripts. We de novo assembled 96,562 high quality unigenes with an average length of 832 bp. A total of 33,662 full-length ORF complete sequences were identified, and 41,415 unigenes were mapped onto 128 pathways using the KEGG Pathway database. The annotated unigenes were compared against *Brassica rapa*, *B. oleracea*, *B. napus* and *Arabidopsis thaliana*. Among these unigenes, 76,324 were identified as putative homologs of annotated sequences in the public protein databases, of which 1194 were associated with plant hormone signal transduction and 113 were related to gibberellin homeostasis/signaling. Unigenes that did not match any of those sequence datasets were considered to be unique to *S. arvensis*. Furthermore, 21,321 simple sequence repeats were found. Our study will enhance the currently available resources for Brassicaceae and will provide a platform for future genomic studies for genetic improvement of Brassica crops.

## Introduction

The Brassicaceae comprises 49 tribes, 321 genera and 3660 species and is one of the most important crop plant families [1]. *Sinapis arvensis* is an annual or winter annual plant of the genus Sinapis [2]. *S. arvensis* is frequently found growing in *Brassica napus* and *Triticum aestivum* fields and is thought a kind of “poisonous weeds” for agricultural production [3]. As a weed, it has strong environmental adaptability to various biotic and abiotic stresses. In Canada, the hybridization of transgenic oilseed rape with *Sinapis arvensis* is a potential genetic risk [4]. *S. arvensis* has recently become an available germplasm resource for rapeseed breeding. The superior traits of *S. arvensis* such as resistance to drought, pod shatter, blackleg and stem rot diseases, low contents of erucic acid and sulfuric glycoside as well as cytoplasmic male sterility and its restoration were introduced into rapeseed via hybridization or protoplast fusion [5]–[7]. So far, the detailed genome analyses in Brassicaceae have been almost exclusively limited to the model plant Arabidopsis and the leading crop species of *Brassica rapa* [8], *B. oleracea* and *B. napus* (unpublished). However, the genome information about wild species like *S. arvensis* is still fragmentary or non-existent. *S. arvensis* is a “diploid” species, belonging to the nigra lineage (B genome) as determined from phylogenetic relationships and the history of polyploidy in Brassica [9]. In contrast to the Brassica A and C genomes, the B genome requires in-depth study since it possesses important agronomic traits for canola breeding.

The advancement in high-throughput sequencing technology provides us the opportunity to decipher valuable genomic information about these non-model plants [10]. The deep coverage and single base-pair resolution allows the use of RNA-Seq as a powerful and cost-efficient tool for advanced research in mRNA, non-coding RNA and small RNAs of a plant. These studies help to determine the transcriptional structure of genes and quantify the changing expression levels of each transcript during development and other conditions [11]. Furthermore, it helps in the identification of genetic markers such as microsatellites and single nucleotide polymorphisms (SNPs) [12]. Several studies have used RNA-Seq approaches in Brassicaceae plants during the past five years, particularly in the model plant Arabidopsis and leading crop species of Brassica [13]–[15], Thellungiella [16] and Raphanus [17]. More EST sequences and well-assembled transcriptome sequences would help in the advancement of research in the Brassicaceae family. The advent of high-throughput sequencing technologies enables generation of genomic resources in a short time at a minimal cost, and therefore provides a turning point for Sinapis research.

In order to provide well-assembled transcriptome sequences of *S. arvensis* to the Brassicaceae (cruciferous) research community, we perform the *de novo* transcriptome sequencing of *S. arvensis*. Flowers of *S. arvensis* were used for high-throughput sequencing based on the Illumina Hiseq 2000 platform. We collected over 39,586,590 clean reads and assembled them into 96,562 unigenes. Annotation and gene ontology analysis were performed on these unigenes, providing a valuable resource for future genetic and genomic research in *S. arvensis* as well as closely related species.

## Results

### Sequence analysis and assembly

To obtain a global overview of the *S. arvensis* transcriptome and gene activity at nucleotide resolution, a mixed cDNA sample representing different flower developmental stages of *S. arvensis* was prepared and sequenced using the Illumina Hiseq 2000. The sequenced sample yielded 2×100-bp independent reads from either end of a cDNA fragment. After stringent quality assessment and data filtering, 40,981,443 reads (∼8.2 G) with 97% Q20 bases (those with a base error rate lower than 1%) were selected as high quality reads for further analysis.

**Figure 1:**
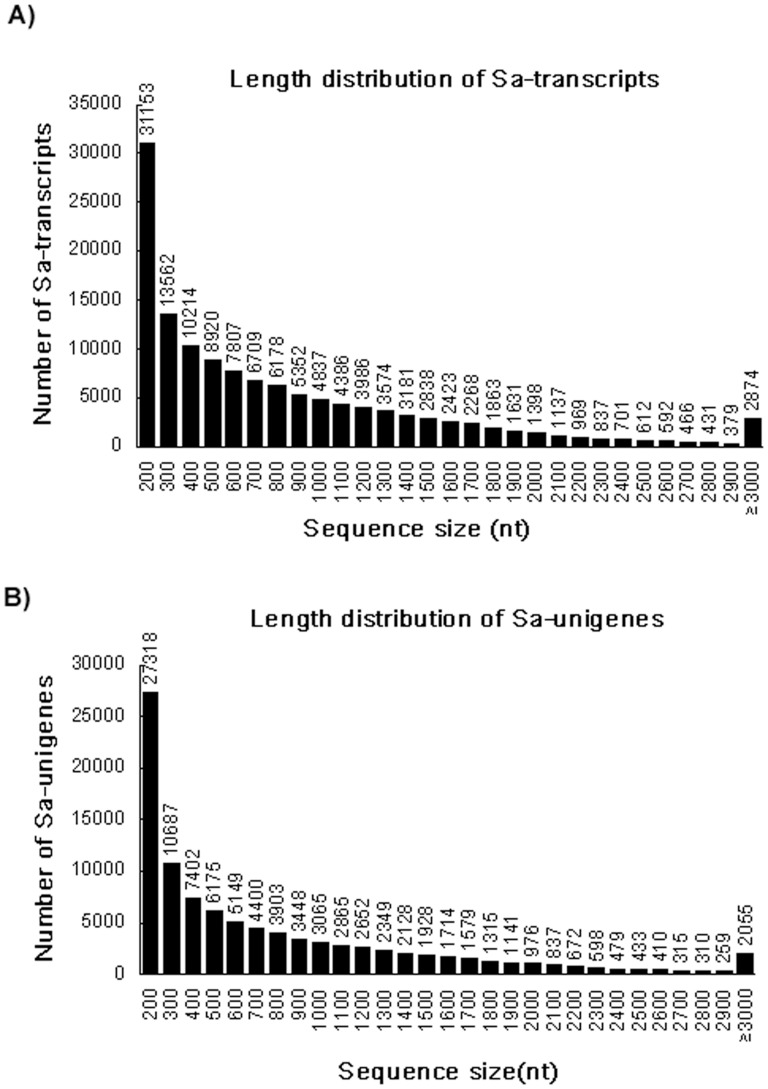
Size distribution of *S. arvensis* unigenes and transcripts.

Using the Trinity *de novo* assembly program, short-read sequences were assembled into 131,278 transcripts, with a N50 length of 1,308 bp and an average length of 877 bp (Table 1). Overall, there were 41,383 scaffolds coding for transcripts longer than 1 kb and 10,396 scaffolds coding for transcripts longer than 2 kb (Figure 1A). The transcripts were subjected to cluster and assembly analyses. A total of 96,562 unigenes were obtained, among which 28,080 genes (29.08%) were greater than 1kb. The length distribution of unigenes revealed that more than 51,155 unigenes (∼53.0%) were greater than 500 bp (Figure 1B). These results demonstrated the efficacy of Illumina pyrosequencing in rapidly capturing a large portion of the transcriptome. As expected for a randomly fragmented transcriptome, there was a positive relationship between the length of a given unigene and the number of reads assembled into it. Above all, these results demonstrated the reliability of Illumina paired-end sequencing and *de novo* assembly.

**Table 1:** Summary of *Sinapis arvensis* transcriptome assembly.

Assembly statistics	
Total number of paired-end reads (before trimming)	40,981,443
Total number of read base pairs (bp)	8,196,288,600
Average read length (before trimming; bp)	100
Total number of paired-end reads (after trimming)	39,586,590
Total number of transcripts assembled (pre-isoform filtering)	131,278
Total number of transcripts Length (bp)	115,066,714
Average length of all transcripts (bp)	877
N50 of all transcripts	1,308
Total number of unigenes assembled (post-isoform filtering)	96,562
Total number of unigenes Length (bp)	80,370,734
Average length of all unigenes (bp)	832
N50 of all unigenes	1,307

### Sequence annotation

We utilized several complementary approaches to annotate the assembled sequences. The unigenes were annotated by aligning with the deposited ones in diverse protein databases such as National Center for Biotechnology Information (NCBI), non-redundant protein (Nr) database, NCBI non-redundant nucleotide sequence (Nt) database, Swiss-Prot, Kyoto Encyclopedia of Genes and Genomes (KEGG) and Cluster of Orthologous Groups of proteins (COG). First, a sequence similarity search was conducted against the NCBI Nr and Nt database and Swiss-Prot protein database using the Basic Local Alignment Search Tool (BLAST) algorithm specifying E-values of less than 10^−5^. The analysis indicated that 76,324 (79.0%) of the 96,562 unigenes had significant matches in the Nr database, 83,216 (86.2%) had significant matches in the Nt database, and 50,064 (51.8%) had similarity to proteins in the Swiss-Prot database.

**Figure 2:**
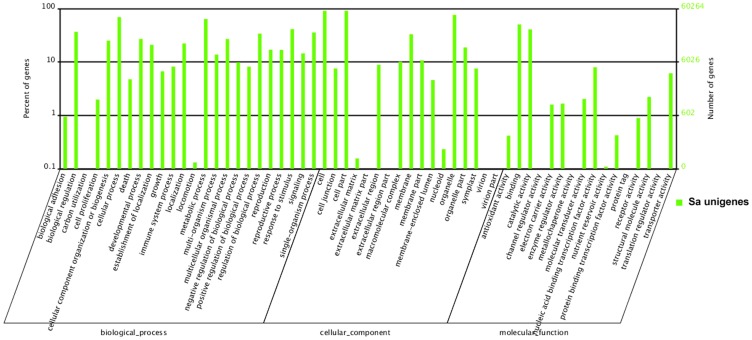
Gene Ontology (GO) analysis of transcriptome associated with flower development of *S. arvensis*. (The percentage and distribution of top-level GO-terms were portrayed in the three categories: Biological process, Cellular component and Molecular function.). Gene Ontology classifications of assembled unigenes, and 76,324 unigenes with significant similarity in Nr protein databases were assigned to gene ontology classifications.

Gene Ontology (GO) analysis was carried out based on the Nr notation. It provided a dynamic, controlled vocabulary and hierarchical relationships for the information on molecular functions, cellular components and biological processes, allowing a coherent annotation of gene products. Among the 76,324 unigenes annotated in Nr, one or more GO terms were assigned to the 60,264 unigenes, with 51.6% for biological processes, 36.7% for molecular functions and 11.7% for cellular components (Figure 2). For biological processes, the genes involved in cellular process (GO: 0009987) and metabolic process (GO: 0008152) were highly represented. For molecular functions, binding activity (GO: 0005488) was the most represented GO term, followed by the catalytic activity (GO: 0003824). The most represented category for cellular components was cells (GO: 0005623) (Figure 2).

**Figure 3:**
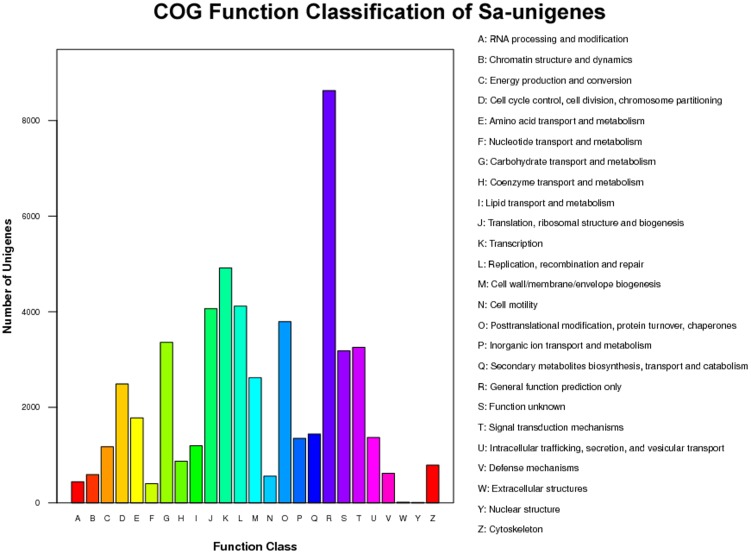
Clusters of orthologous groups (COG) classification. In total, 25,512 of the 60,264 sequences with Nr hits were grouped into 25 COG classifications.

Furthermore, all unigenes were subjected to a search against the COG database [18] for functional prediction and classification. Overall, 25,515 of the 60,264 sequences showing a hit within the Nr database were assigned to COG classifications (Figure 3). COG annotated putative proteins were functionally classified into at least 25 protein families involved in cellular structure, biochemistry metabolism, molecular processing, signal transduction and so on (Figure 3). The cluster for general function prediction (2,673; 26.34%) represented the largest group, followed by replication, recombination and repair (1,359; 13.39%), transcription (1,319; 13%), signal transduction mechanisms (1,096; 10.8%), translation, ribosomal structure and biogenesis (1,004; 9.89%), post-translational modification, protein turnover and chaperones (964; 9.5%), carbohydrate transport and metabolism (831; 8.19%), amino acid transport and metabolism (673; 6.63%), energy production and conversion (538; 5.3%). On the other hand, only a few unigenes were assigned to nuclear structure and extracellular structure (18 and 5 unigenes, respectively). In addition, 368 unigenes were assigned to cell wall/membrane/envelope biogenesis, and 248 unigenes were assigned to intracellular trafficking, secretion and vesicular transport (Figure 3).

To demonstrate the usefulness of *S. arvensis* unigenes generated in the present study further, we identified biochemical pathways represented by the unigene collection. Annotations of *S. arvensis* unigenes were fed into the KEGG Pathway Tools [19], which is an alternative approach to categorize gene functions with an emphasis on biochemical pathways (Table S1). This process predicted a total of 128 pathways represented by a total of 20,635 unigenes. Summary of the sequences involved in these pathways is included in Table S2. These predicted pathways represented the majority of plant biochemical pathways for the compound biosynthesis, degradation, utilization, and assimilation, and pathways involved in the processes of detoxification and generation of precursor metabolites and energy. Enzymes catalyzing almost all steps in several major plant metabolic pathways including the Calvin cycle, glycolysis, gluconeogenesis, the pentose phosphate pathway and several important secondary metabolite biosynthesis pathways were completely included. Interestingly, 1194 genes (5.79%) associated with the plant hormone signal transduction (ko04075) were found to be involved in flower development. Moreover, genes involved in several signaling pathways, such as plant-pathogen interaction, natural killer cell mediated cytotoxicity and circadian rhythm were also found in the unigene collection.

### Comparative analysis with *Brassica rapa* and other members of the family Brassicaceae

**Figure 4:**
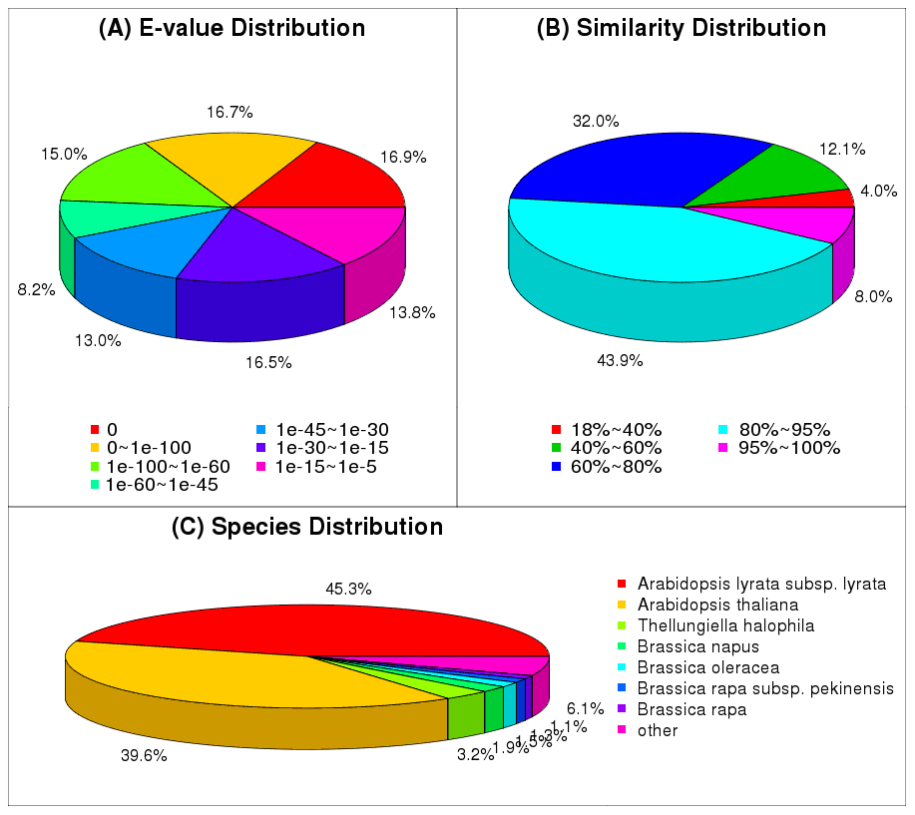
Statistics of homology search of unigenes against nr peptide database. A) E-value distribution of the top BLASTx hits with a cut-off E-value of 1e-05. B) Similarity distribution of the top BLASTx hits with a cut-off E-value of 1e-05. C) Species distribution of the top BLASTx hits is shown as a percentage of the total homologous sequences with an E-value greater than or equal to 1e-05.

We did a new BLAST operation to study the relationship of *S. arvensis* with other Brassicaceae members in terms of orthology or to identify proteins or pathways that might be unique to *S. arvensis*. The unigene set of *S. arvensis* was analyzed for similarity and/or sequence conservation against the gene data sets of various Brassicaceae species namely *Arabidopsis lyrata*, *Arabidopsis thaliana*, *Thellungiella halophila*, *Brassica napus, Brassica oleracea* and *Brassica rapa* using TBLASTX search. An *E*-value cut-off threshold of ≤1e^−05^ was considered to define a significant hit. *S. arvensis* unigenes showed significant similarity mostly with *Arabidopsis lyrata* unigenes (45.3%) followed by *Arabidopsis thaliana* (39.6%), *Thellungiella halophila* (3.2%), *Brassica napus* (1.9%), *Brassica oleracea* (1.5%) and the least similarity with *Brassica rapa* (1.1%; Fig. 4C). Overall, a total of 76,324 (79.0%) of the *S. arvensis* unigenes showed significant similarity to at least one of the Brassicaceae genes (Fig. 4A). Likewise, we analyzed the sequence conservation of *S. arvensis* unigenes with proteomes of all sequenced plant species. Although a large number of the *S. arvensis* unigenes showed significant similarity with predicted proteins from Brassicaceae members, the extent of coverage of the coding region was a little more than expected. 83.9% of the unigenes showing significant similarity covered ≥60% of the coding region of the predicted proteins from other Brassicaceae, including 8.0% of unigenes with 95%–100%; 43.9% with 80%–95% and 32.0% with 60%–80% similarity (Fig. 4B), respectively. Considering the high degree of conservation among members of Brassicaceae, it can be assumed that the assembly of *S. arvensis* transcriptome could be further improved as more sequence data become available.

Further, the combined analysis of BLAST results with Brassicaceae unigenes and plant proteomes revealed that a total of 76,324 (79.0%) unigenes were conserved in *S. arvensis* showing significant similarity with at least one sequence. Besides 83,216 unigenes had significant matches in the Nt database, 13,346 (13.8%) unigenes did not show significant similarity with any of the data set analyzed, which probably represent *S. arvensis* -specific genes.

### GA homeostasis/signaling genes involved in flower development

**Figure 5:**
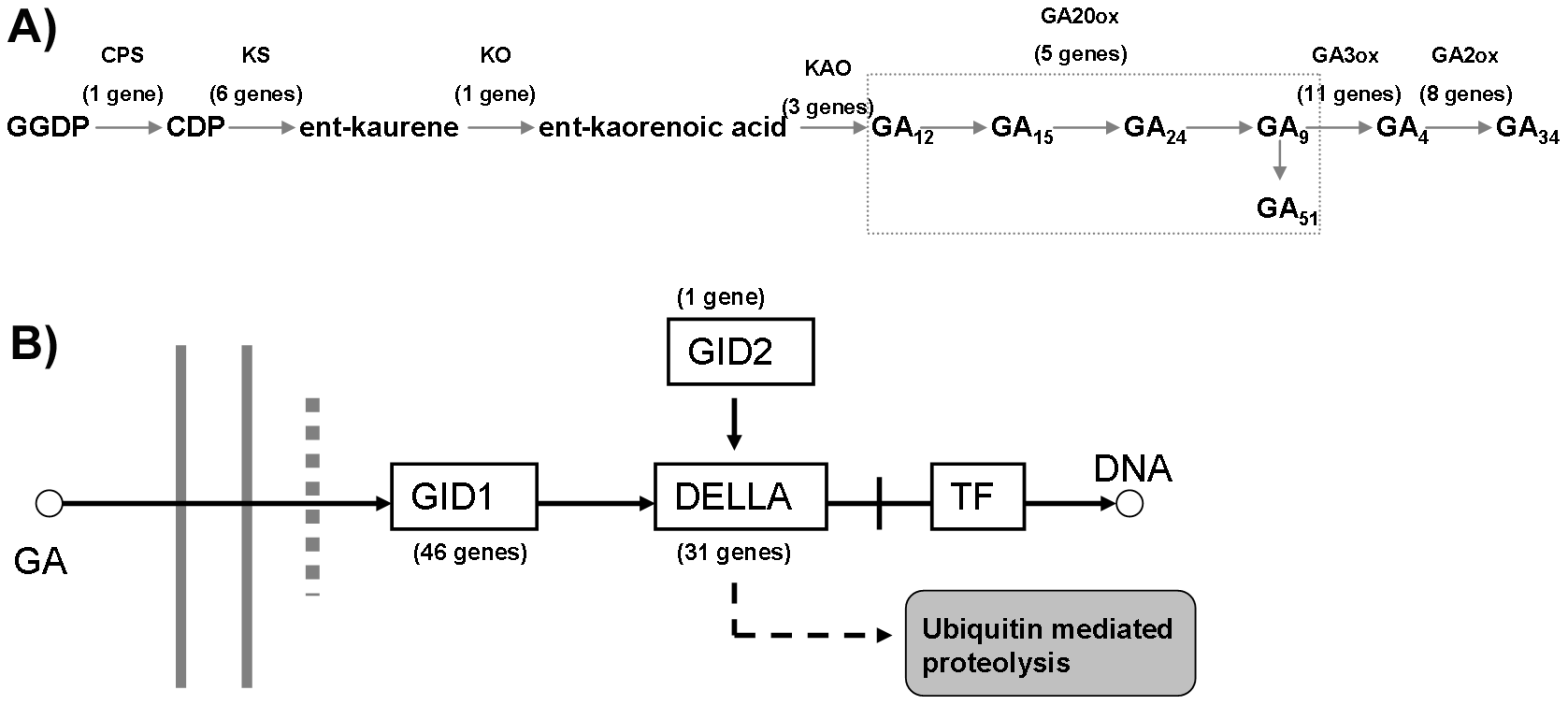
Unigenes of GA Biosynthetic (A) and signal transduction (B) found in *S. arvensis*. This is a simplified diagram only showing the pathways for the synthesis of non-13-hydroxylated GAs. Gray text indicates GA synthetic and catabolic enzymes catalyzing corresponding steps. GGDP, geranylgeranyl diphosphate; CDP, ent-copalyl diphosphate; CPS, ent-copalyl diphosphate synthase; KS, ent-kaurene synthase; KO, ent-kaurene oxidase; KAO, ent-kaurenoic acid oxidase.

By KEGG clustering of the unigenes from *S. arvensis* flower, we found that a total of 35 genes involving in GA synthesis and 78 genes involving in GA signal transduction (Figure 5 and Table S2). Genes involved in the entire GA homeostasis/signaling network were captured. GA synthesis can be classified into early and late stages, with CPS (ent-copalyl diphosphate synthase) encoded by GA1 acting in early synthesis and GA20-oxidase and GA3-oxidase in later steps (Figure 5A). In GA synthesis processor, except KS (6 unigenes), CPS (1 unigene), KO (1 unigene) and KAO (3 unigenes) had a relatively conserved function. However, downstream synthesis genes GA20ox, GA3ox and GA2ox had higher expression levels, and the homologous genes were more abundant. Therefore, GA catabolism by GA oxidases may regulate tissue-specific pools of active GA in floral development. The GA signal transduction genes GID1 (46 unigenes) and DELLA (31 unigenes) were identified (Figure 5B), both of which have a large number of homologous genes involved in flower development. GID2 showed a higher expression level as compared to GID1 and DELLA and was the only unigene that was involved in the suppression of flower development.

### Full-length cDNA prediction

Full-length cDNA sequences are important in genetics and genomics research, such as for gene duplication analysis, alternative splicing, whole genome sequencing and assembly, etc. TargetIdentifier [20] was used to analyze the transcripts to identify potential full-length cDNAs with a complete ORF in the assembled transcriptome of *S. arvensis*. A total of 33,662 full-length and completely ORF sequences were identified from the assembly with a cutoff E-value of 1e^−5^ (Table 2). TargetIdentifier predicted that there were 56,880 full-length, 6,748 short full-length, 22,547 5′-partial, 21,787 3′- partial and 897 ambiguous sequences in the *S. arvensis* transcripts pool. The result proved that *de novo* assembly of Trinity was very efficient in recovering full-length transcripts and spliced isoforms.

**Table 2:** Targetidentifier results.

Result item	Numbers	Percentage (%)
total number of sequences	131,278	
number of no hit	22,419	17.08
number of sequences with hit	108,859	82.92
full-length sequences	56,880	43.33
short full-length sequences	6,748	5.14
ambiguous sequences	897	0.68
partial sequences (5'-sequenced partial)	22,547	17.18
3'-sequenced partial sequences	21,787	16.60
full-length & ORF completely sequenced sequences	33,662	25.64

### Repetitive element analysis and microsatellite identification

Altogether, 25,801 sequences containing 37,752 potential EST-SSRs were identified from 96,562 consensus unigenes. A total of 21,321 microsatellites were analyzed after removing 16, 431 mono-nucleotide repeats. The most abundant type of repeat motif was tri-nucleotide (53.85%), followed by di-nucleotide (45.08%), tetra-nucleotide (0.88%), pentanucleotide (0.10%), and hexa-nucleotide (0.09%) repeat units. Within the detected EST-SSRs, 164 motif sequence types were identified, of which, di-, tri-, tetra-, penta- and hexa-nucleotide repeats had 3, 10, 21, 10 and 8 types, respectively. Among these, AG/CT (37.66%) represented the dominant type, followed by AAG/CTT (16.55%), AGG/CCT (8.85%), AAC/GTT (6.35%), ACC/GGT (5.46%), ACT/AGT (5.22%), and AC/GT (4.31%) (Table 3). The frequency of the remaining 156 types of motifs accounted for 15.60% repeat units.

**Table 3:** Summary of simple sequence repeat (SSR) types in the *S. arvensis* transcriptome.

Repeat motif	Number	Percentage (%)	
**Di-nucleotide**			
AC	920	4.31	
AG	8,030	37.66	
AT	662	3.10	
**Total**	**9,612**		**45.07**
**Tri-nucleotide**			
AAC	1,353	6.35	
AAG	3,529	16.55	
AAT	200	0.94	
ACC	1,164	5.46	
ACG	578	2.71	
ACT	1,113	5.22	
AGC	452	2.12	
AGG	1,886	8.85	
AGT	916	4.30	
CCG	290	1.36	
**Total**	**11,481**		**53.85**
**Tetra-nucleotide**			
AAAC/AAAG/AAAT	75	0.35	
AACC/AACG/AACT/AAGC/AAGG/AAGT/AATC/AATG	72	0.34	
ACAG/ACAT/ACCT/ACGC/ACGT/ACTC	23	0.11	
AGAT/AGCC/AGCG/AGGG	17	0.08	
**Total**	**187**		**0.88**
**Penta-nucleotide**			
AAAAT/AAACC/AAAGG	3	0.01	
AACTT/AAGCT/AAGGG	12	0.06	
ACATC/ACTCC/AGAGC/AGAGG	7	0.03	
**Total**	**22**		**0.10**
**Hexa-nucleotide**			
AAAACC/AAACGG/AAAGAG	10	0.05	
AAGACG/AAGATG/AAGGAG/AAGGCC/ACACCC	9	0.04	
**Total**	**19**		**0.09**

## Discussion

Our *de novo* analysis identified 96,562 unigenes expressed in the development of flower buds. These unigenes have been shown to be particularly useful for obtaining gene sequence information of non-model crops such as radish, grape, sunflower, etc [21]. We identified unigenes specific to *S. arvensis* by comparing its transcriptome data to other genomes. Certain genes that have been previously shown to be essential for floral development and reported to be tissue-specific in other plants were also discovered in our study.

Gibberellin (GA) plays an important role in regulating many aspects of flower development. Although molecular function research on GA in Arabidopsis is of great significance, there is limited availability of this data from other plants. Several homologous genes or multiple copies of genes involved in GA synthesis and transduction were identified using RNA-seq. Consequently, the methods that can simultaneously analyze multiple RNA processing events are culminating in the development of genome-wide RNA maps that pave the way for future research. The GA genes involved in flower development provide a comprehensive understanding of the developmental process. A number of genes, including those coding for enzymes at different points in GA biosynthesis, have been characterized. The study of the floral expression patterns and mutant phenotypes of these genes allows inferences to be made about biologically active GA pools during flower development. This is especially important as currently little is known about how plants regulate GA levels developmentally. In Arabidopsis, GA1 expression is high in IMs, early floral primordia, the receptacle, mature anthers, and pollen [22]. The Arabidopsis genes, GA20-oxidases, GA20ox1 and GA20ox2 play redundant roles in stamen filament elongation and fertility, whereas GA20ox4 and GA20ox5 are weakly expressed in mature pollen [23]. GA is essential for the development of stamens. *De novo* GA synthesis and bioactive GAs synthesized in the stamens and/or flower receptacles and transported to petals are necessary for early stamen development [24]. The role of GAs in regulating fertility, petal growth, and stamen filament elongation via cell elongation is countered by the repression of DELLA protein [25]. The identification of unigenes coding for enzymes and the related unigenes involved in GA biosynthesis and transduction pathways will help in facilitating floral development and hormone-related research of *S. arvensis*, which in turn would play a key role on further study of a new cytoplasmic male sterilty system derived from somatic hybridization between *B. napus* and *S. arvensis* [5].

Simple sequence repeat (SSR) markers are useful for a variety of applications in plant genetics and breeding because of their genetic co-dominance, abundance, dispersal throughout the genome, multi-allelic variation, high reproducibility and high level of polymorphism [26]. SSRs can be divided into genomic SSRs and EST-SSRs, on the basis of the original sequences used to identify simple repeats. EST-SSRs are derived from expressed sequences, which are more evolutionarily conserved than non-coding sequences; therefore, EST-SSR markers have a relatively higher transferability than genomic SSRs [27]. Furthermore, the traditional methods used to isolate and identify genomic SSRs are costly, labor intensive and time-consuming.

The mapped reads of *S. arvensis* bud transcriptome yielded 21,321 potential SSRs. Thus far, a number of EST-SSR markers have been developed for genetic and genomic studies and to enhance breeding programs in Brassicaceae [28], [29]. We analyzed the SSR frequency distribution for each mapped unigene and the possibility of mapping putative SSRs to closely related paralogues. The identification of species-diverged DNA fragments is of considerable significance in Brassica research, particularly in identifying the authenticity of hybrids and distinguishing specific chromosomes [30]. This study provided us with the information related to discovery of numerous SSR markers to be readily used in diversity analysis in Brassicaceae. Moreover, QTL mapping and breeding programs involving new rapeseed lines derived from *S. arvensis* will benefit from the SSR markers discovered as a result of our deep-sequencing data. The total potential SSRs discovered can be filtered in order to obtain the species-specific SSRs in the related species of Brassicaceae. The partial genome of *S. arvensis* with pod shatter and sclerotinia resistant genes was imported into the Brassica genome [7]. Our study will provide a better platform for future such genetic modifications in Brassica species.

## Conclusion

Our results present the first broad survey of a *S. arvensis* flower transcriptome. Although it will be necessary to validate the functions carried out by these genes, our results represent a starting point for future functional research on *S. arvensis* and related species. This report is the first to identify all unigenes associated with GA biosynthesis and transduction in the flower bud and represents a valuable resource for future genomic studies in *S. arvensis*. Thus, our deep sequencing study not only enriched the publicly available database for Brassicaceae, it will help in making economically important hybrids in the family with desirable genetic traits.

## Materials and Methods

### Sample preparation and RNA isolation

*Sinapise arvensis* cv. “Yeyou18” was collected from Xinjiang, China. Since it is a wild germplasm, we planted it over ten generations and bagged the plants to obtain pure seeds. The plants were grown in a controlled illuminated culture room of Oil Crops Research Institute at 23±1°C/19±1°C day (14 h)/night (10 h) with a photosynthetic photon flux density of approximately 300–320 µmol m^−2^ s^−1^. Flower buds were collected at three sizes based on buds length. The phase 1 sample consisted of buds ≤1.5 mm; the phase 2 sample consisted of buds ≥ 1.5 mm and ≤ 3 mm, and the phase 3 consisted of buds ≥ 3 mm. Samples were immediately frozen in liquid nitrogen and stored at −80°C until RNA was extracted. The total RNA of each sample was isolated using RNAprep pure Plant Kit (TIANGEN). RNA quality was characterized using an agarose gel and the NanoDrop ND1000 spectrophotometer (NanoDrop Technologies, Wilmington, DE, USA) and was further assessed by RIN (RNA Integrity Number) value (>9.5) using an Agilent 2100 Bioanalyzer (Santa Clara, CA, USA). Equal quantities of high-quality RNA from each material were pooled for cDNA synthesis.

### cDNA library construction for Illumina sequencing

A cDNA library was constructed using the mRNA-Seq Sample Preparation Kit (Cat*#* RS-930-1001, Illumina Inc, San Diego, CA) (Illumina) following the manufacturer’s instructions. Briefly, the poly-(A) mRNA was isolated from the total RNA samples with Magnetic Oligo (dT) Beads. The mRNA was then fragmented into small pieces using an RNA fragmentation kit (Ambion). Using these short fragments as templates, the first cDNA strand was synthesized using random hexamer primers and reverse transcriptase (Invitrogen), and the second-strand cDNA was synthesized using DNA polymerase I and RNase H. The cDNA fragments were purified using the QiaQuick PCR extraction kit (Qiagen) and resolved with EB buffer for end reparation and poly(A) addition. The short fragments were then connected with sequencing adapters, and the products were subsequently purified and amplified via PCR. Libraries were prepared from a 400–500 bp size-selected fraction following adapter ligation and agarose gel separation. A quality control analysis on the sample library was performed to quantify the DNA concentration and to validate the library. After validation with an Eppendorf Mastercycler ep realplex Real-Time PCR System, the cDNA libraries were sequenced on the Illumina Hiseq 2000 platform. The sequencing-derived raw image data were transformed by base calling into sequence data using the Illumina Pipeline Software v1.6.

### Sequence data analysis and assembly

The raw reads were cleaned by removing the adapter sequences; low-quality sequences (reads with ambiguous bases ‘N’) and reads with more than 10% Q<20 bases. The high-quality reads were assembled into unigenes with Trinity, which recovers more full length transcripts across a broad range of expression levels, with sensitivity similar to methods that rely on genome alignments [31]. Contigs without ambiguous bases were obtained by conjoining the K-mers in an unambiguous path. Thereafter, we used Trinity to map the reads back to contigs to construct unigenes with the paired-end information. The program detected contigs from the same transcript as well as the distances between these contigs. Next, the contigs were connected with Trinity, and the sequences that could not be extended on either ends were obtained. Such sequences are defined as unigenes. Finally, the overlapping unigenes were assembled into a continuous sequence using the overlapping ends of different sequences.

### Functional annotation

Sequence directions of the resulting unigenes was determined by performing BLASTX searches against protein databases, with a priority order of Nr (non-redundant protein sequences in NCBI), Swiss-Prot, Kyoto Encyclopedia of Genes and Genomes database (KEGG), and COG (E-value ≤ 1e-5) if conflicting results were obtained.

Local BLASTx was performed to align assembled transcriptome unigenes to NCBI non-redundant (nr) protein database for functional annotation. The e-value cutoff was set at 1e-5. Gene name was assigned to each contig based on the top BLASTx hit with the highest score. We searched all six-frame translations of unigenes against the Nr plant protein database in NCBI (by running BLASTX with an E-value cut-off 1e-5) to compare *S. arvensis* unigenes were compared with the currently available genome sequences from the Nr database.

Gene ontology (GO) analysis was conducted on the assembled transcriptome sequences by using InterProScan (http://www.ebi.ac.uk/Tools/pfa/iprscan/) and integrated protein databases with default parameters. The GO terms associated with each assembled sequence of *S. arvensis* transcriptome were then obtained for describing the biological processes, molecular functions and cellular components.

To evaluate the completeness of our transcriptome library and the effectiveness of our annotation process, we searched the annotated unigene sequences for the possible functions involved in COG classifications. Furthermore, to summarize the active pathways in *S. arvensis* flowers, KEGG pathways were assigned to assemble contigs using the online KEGG Automatic Annotation Server (KAAS) (http://www.genome.jp/tools/kaas/) [32]. The Bi-directional Best Hit (BBH) method was used to obtain KEGG Orthology (KO) assignment. The remaining unigenes were analyzed by ESTscan to search for CDS, which could be used to distinguish coding and non-coding sequences [33]. ESTScan software was also used to determine the direction of sequences that were not aligned to sequences in any of the databases mentioned above.

### Full-length cDNA identification

Putative full-length cDNAs were identified by using the online tool TargetIdentifier [20], which were compared to non-redundant protein databases with a cutoff e-value of 10^−5^. The cDNA sequence was recognized as a full-length cDNA only if the start codon (ATG) and poly (A) tail were identified. According to the definition of TargetIdentify prediction, each unigene sequence was classified into all-length, short full-length, possible-full length, ambiguous, partial or 3′-sequenced partial cDNA.

### Repetitive element analysis, microsatellite identification

To identify all repetitive elements in the assembled transcriptome of *S. arvensis*, the MicroSAtellite software (MISA; http://pgrc.ipk-gatersleben.de/misa/) was used for all assembled unigenes. The mononucleotide repeats were ignored by modifying the configure file. The parameters were adjusted for identification of perfect di-, tri-, tetra-, penta-, and hexa-nucleotide motifs with a minimum of 6, 5, 5, 5 and 5 repeats, respectively.

### Data deposition

The Illumina pair-end reads of *S. arvensis* obtained in the study are now available at NCBI SRR1066782.

## Supporting Information

Table S1 **KEGG biochemical mappings for** ***S. arvensis*** **.**
(XLS)

Table S2 **Unigenes information of GA Biosynthetic and signal transduction in** ***S. arvensis*** **flower.**
(XLS)
